# Superior Thyroid Cornu Anatomical Variation Causing Globus Pharyngeous and Dysphagia

**DOI:** 10.1155/2010/142928

**Published:** 2010-11-11

**Authors:** Jiannis K. Hajiioannou, Vasiliki Florou, Panagiotis Kousoulis

**Affiliations:** General Hospital of Nikea, D. Mantouvalou 3, 18454 Nikea, Piraeus, Greece

## Abstract

*Objective*. Rare case presentation of thyroid cartilage variation causing globus sensation and dysphagia. *Method*. Case report and review of the literature concerning thyroid cartilage variant of clinical significance. *Results*. Middle-aged male patient presented with globus sensation and painful swallow without previous injury of the larynx. Clinical examination and diagnostic procedures revealed a rare anatomic aberrance of the thyroid cartilage. Surgical treatment was declined by the patient who accepted a yearly followup. *Conclusion*. Morphometric studies do not report the dislocation of the superior thyroid cornu, and very few cases have been described either of which were attributed to trauma or to unknown cause. The present case is to be added to the very few cases of superior thyroid cornu dislocation of unknown aetiology. Clinicians should be aware of this rare variation using CT neck as the imaging study of choice. Direct endoscopy rules out any synchronous disease or malignancies.

## 1. Introduction

Dysphagia is the difficulty in swallowing and may be attributed to oral, pharyngeal, or oesophageal dysfunction. Common patient's complains include globus pharyngeous, “sticking of food” in the upper neck, suprasternal or substernal area, nasal regurgitation, failure of swallowing initiation, aspiration, pain-odynophagia, and heartburn [1]. 

Causes of swallowing disorders can be congenital or acquired, and the latter include trauma, infections, inflammations, esophageal motility disorders, neoplasms, neurologic disorders, medication, age (presbydysphagia), foreign body, caustic lesions, pharyngeal pouch, globus pharyngeous, tracheostomy, and thyroid disease [2].

We present a case of globus pharyngeous and dysphagia due to rare anatomical variation of the thyroid cartilage.

## 2. Case Report

A 56-year-old male attended the ENT outpatient Clinic complaining of foreign body sensation in the neck and intermittent painful swallow. Symptoms had appeared 3 months ago without history of neck trauma or other incident. On examination, the patient appeared malnourished and reported increased alcohol consumption. He was a heavy smoker and reported a history of hypertension and angina. 

**Figure 1:**
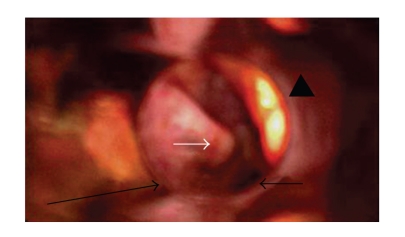
EUA. Direct hypopharyngoscopy, white arrow shows the bulge of the posterior hypopharyngeal wall into left piriform sinus. The long black arrow shows the posterior pharyngeal wall while the head of an arrow demonstrates the epiglottis. The short black arrow shows small part of the glottis marginally visible through the direct endoscope.

Routine ENT examination was unremarkable, as was the flexible endoscopy of the larynx. A barium swallow test also did not show any pathology, and the patient was scheduled for examination under anaesthesia (EUA) which included direct hypopharyngoscopy, microlaryngoscopy, and esophagoscopy. The EUA was normal for esophagus and larynx, but revealed a bulge of the left posterior hypopharyngeal wall protruding into the left piriform sinus. On palpation, it was of bony or cartilage consistency, it had no characters of malignancy and was lined by smooth, macroscopically healthy mucosa (Figure 1). It was suggested that thyroid cornu could be the underlying cause of the lesion, and a CT scan of the neck was ordered as the imaging study of choice. Biopsy was not an option as the underlying tissue was unknown.

**Figure 2:**
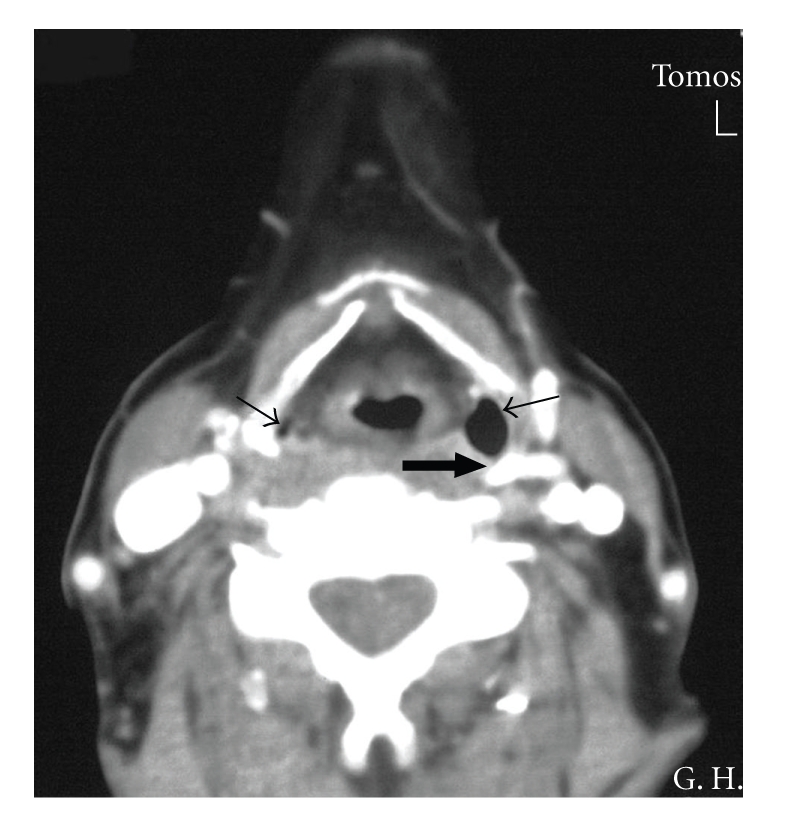
CT neck. Axial cut with contrast at the level of C5 vertebra. Rotation of thyroid cartilage, black arrow shows the left thyroid cornu. The airfilled cavity next to the left thyroid cornu is the left piriform sinus which is clearly bigger than the right one due to the rotation of the larynx (black thin arrows).

CT scan confirmed the diagnosis of rotated thyroid cartilage and dislocation of the left superior thyroid cornu with medial anterior projection into the ipsilateral piriform sinus (Figure 2). 

The patient was reassured about the benignity of the disease and declined any further surgical treatment. However, he was advised to attend a yearly followup, due to the rest of his history.

## 3. Discussion

The thyroid cartilage is the framework of the larynx presenting two superior and two inferior cornua. Lateral thyrohyoid ligament is attached to the superior cornu extending to the greater hyoid cornu. Hirano et al. [3] studied cadaveric thyroid cartilages noticing that in older adults; there was a directional preponderance in asymmetry. The thyroid cartilage as a whole tended to tilt to the right against the cricoid cartilage. Later morphometric measurements of the laryngeal framework provided valuable information determining the size and extent of the cartilaginous components and human larynx as one unit [4, 5]. However, no remarks were done regarding any superior cornu dislocation. 

The dislocation of superior thyroid cornu was first described in 1994 by Avrahami et al. [6], as a result of laryngeal trauma of inflexible ossified laryngeal cartilages. Later in 2001, Smith et al. [7] presented a series of 11 cases of posttraumatic throat pain caused by swallowing or neck rotation, mostly due to superior cornu of the thyroid cartilage projecting posteriorly and medially. 

Only recently in 2000, the first series of 5 cases with similar anatomic variation, not attributed to trauma, were reported by Browning and Whittet. Resection of the cornu tip protruding in the hypopharynx led to resolution of symptoms [8]. Since that study, 3 more papers have been published in the years 2005 and 2006 describing the presence of this anomaly without previous neck injury, intubation or other manipulation in the neck or larynx [9–11]. 

Various theories tried to address the issue. Ossification of thyroid cartilage starts at the age of 20 to 23 at the inferior margin extending posteriorly at each ala, but superior margin is never ossified [12]. However, an altered ossification of thyroid cartilage could possibly explain the dislocation of the superior cornu and the delayed onset of globus or dysphagia. It was also suggested that this could be a congenital defect of the 4th arch development, without, however, explaining the presentation of symptoms in older age [8]. There are not many cases reported and, therefore, not much evidence to support any of these theories. We present our case to add our experience to the very few reports of international literature. 

The symptoms of this entity are usually nontypical ranging from mild globus sensation to odynophagia. Consequently, differential diagnosis includes several diseases like gastroesophageal reflux, laryngeal Ca, hypopharyngeal Ca, neurologic disorders, and so forth. 

Our patient's status of malnourishment, smoking, and alcohol consumption mandated further investigations which revealed the rare anatomical variant. Direct endoscopy is essential in excluding malignancy or serious disease; however, CT is the pathognomonic imaging study for depicting these anatomical structures [9]. 

The treatment is surgical, usually endoscopic endolaryngeal resection, but it is frequently denied by the patients as soon as malignancy is ruled out.

## 4. Conclusion

Patients presenting with nontypical symptoms must undergo thorough clinical examination. Differentiation must be made by malignant situations which are presented with similar symptoms and affect patients' survival. Clinicians should be aware of the rare anatomical variant of superior thyroid cornu which despite being benign can be very inconvenient for the patient. Besides, most of the patients are mainly worried about the nature of the disease, and as soon as they are reassured about the benign nature of their condition, they choose to tolerate the symptom over a surgical treatment.
